# Supplementary material for: Structure of the Lifeact–F-actin complex
Source: PLoS Biol. 2020 Nov 20;18(11):e3000925. doi: 10.1371/journal.pbio.3000925 (PMC7717565; doi:10.1371/journal.pbio.3000925)
Supplement: S1 Table — (DOCX) [file pbio.3000925.s001.docx]

**Structure of the Lifeact–F-actin complex**

Alexander Belyy, Felipe Merino, Oleg Sitsel and Stefan Raunser

**S1 Table.** Cryo-EM data collection, refinement and validation statistics

| **Microscopy** |  |
| --- | --- |
| Microscope | Talos Arctica |
| Voltage (kV) | 200 |
| Defocus range (µm) | -0.6 to -3.35 |
| Camera | Falcon III (Linear mode) |
| Pixel size (Å) | 1.21 |
| Total electron dose (e/Å^2^) | 60 |
| Exposure time (s) | 3 |
| Frames per movie | 40 |
| Number of images | 915 (1,415) |
|  |  |
| **3D Refinement** |  |
| Number of helical segments | 223,480 (246,423) |
| Final resolution (Å) | 3.5 |
| Map sharpening (Å^2^) | -50 |
| Helical rise (Å) | 27.3 |
| Helical twist (°) | -167.18 |
|  |  |
| **Atomic model statistics** |  |
| Non-hydrogen atoms | 15575 |
| Molprobity score | 1 |
| Clashscore | 2.23 |
| EMRinger score | 2.75 |
| Bond RMSD (Å) | 0.0167 |
| Angle RMSD (°) | 1.69 |
| Poor rotamers (%) | 0.3 |
| Favored rotamers (%) | 99.7 |
| Ramachandran favored (%) | 98.16 |
| Ramachandran allowed (%) | 1.84 |
| Ramachandran outliers (%) | 0 |
