# Supplementary material for: Structure of the Lifeact–F-actin complex
Source: PLoS Biol. 2020 Nov 20;18(11):e3000925. doi: 10.1371/journal.pbio.3000925 (PMC7717565; doi:10.1371/journal.pbio.3000925)
Supplement: S3 Table — (DOCX) [file pbio.3000925.s003.docx]

**Structure of the Lifeact–F-actin complex**

Alexander Belyy, Felipe Merino, Oleg Sitsel and Stefan Raunser

**S3 Table**. List of primers, strains and plasmids used in this study.

| Bacterial and yeast strains | Description | Reference |
| --- | --- | --- |
| *E. coli* DH5α | F^-^ Φ80*lac*ZΔM15 Δ(*lac*ZYA-*arg*F) U169 *rec*A1 *end*A1 *hsd*R17(r_k_^-^, m_k_^+^) *pho*A *sup*E44 *thi*-1 *gyr*A96 *rel*A1 λ^-^ | Invitrogen |
| *E. coli* BL21 DE3 | F^–^ *ompT hsdS*_B_(r_B_^–^ m_B_^–^) *gal dcm* (DE3) | Novagen |
| *E. coli* BL21 DE3 CodonPlus RIPL | F^–^ *ompT* *hsdS*(r_B_^–^ m_B_^–^) *dcm*^+^ Tet^r^ *gal*λ(DE3) *endA* Hte [*argU proL* Cam^r^] [*argU ileY leuW* Strep/Spec^r^] | Agilent |
| *S. cerevisiae* MH272-3fα | “Wild-type” strain*, ura3, leu2, his3, trp1, ade2* | (55) |
| *S. cerevisiae* SC483 | *S. cerevisiae* MH272-3fα *act1::*LEU2 *+ ACT1*[Ura3] | (38) |
| *S. cerevisiae* SC489 | *S. cerevisiae* MH272-3fα *act1::*LEU2 *+ ACT1*[His3] | (38) |
| *S. cerevisiae* SC690 | *S. cerevisiae* MH272-3fα *act1::*LEU2 *+ ACT1* D25Y/D222G[His3] | (34) |
| *S. cerevisiae* Y446 | *S. cerevisiae* MH272-3fα + empty vector[Ura3] (pESC-Ura) | This study |
| *S. cerevisiae* Y453 | *S. cerevisiae* MH272-3fα + Lifeact-mCherry[Ade] (2477) | This study |
| *S. cerevisiae* Y479 | *S. cerevisiae* MH272-3fα *act1::*LEU2 *+ ACT1* D25Y/D222G[His3] + Lifeact-mCherry[Ade] (2477) | This study |
| *S. cerevisiae* Y484 | *S. cerevisiae* MH272-3fα *act1::*LEU2 *+ ACT1* L349M[His3] (2478)  Strain *S. cerevisiae* SC483 was transformed with plasmid 2478 and passed over 5-FOA to remove WT actin plasmid with Ura3-marker (38) | This study |
| *S. cerevisiae* Y497 | *S. cerevisiae* MH272-3fα *act1::*LEU2 *+ ACT1* L349M[His3] + Lifeact-mCherry[Ade] (2477) | This study |
| *S. cerevisiae* Y510 | *S. cerevisiae* MH272-3fα + Lifeact-MBP[Ura3] (2481) | This study |
| *S. cerevisiae* Y512 | *S. cerevisiae* MH272-3fα + Lifeact L6K-MBP[Ura3] (2482) | This study |
| *S. cerevisiae* Y513 | *S. cerevisiae* MH272-3fα + Lifeact F10A-MBP[Ura3] (2483) | This study |
| *S. cerevisiae* Y514 | *S. cerevisiae* MH272-3fα + Lifeact I13A-MBP[Ura3] (2484) | This study |
| *S. cerevisiae* Y529 | *S. cerevisiae* MH272-3fα *act1::*LEU2 *+ ACT1*[His3] + Lifeact-mCherry[Ade] (2477) | This study |
| *S. cerevisiae* Y552 | *S. cerevisiae* MH272-3fα + Lifeact L6K-mCherry[Ade] (2485) | This study |
| *S. cerevisiae* Y553 | *S. cerevisiae* MH272-3fα + Lifeact F10A-mCherry[Ade] (2486) | This study |
| *S. cerevisiae* Y554 | *S. cerevisiae* MH272-3fα + Lifeact I13A-mCherry[Ade] (2487) | This study |
| *S. cerevisiae* Y555 | *S. cerevisiae* MH272-3fα + Lifeact E17K-mCherry[Ade] (2483) | This study |
| *S. cerevisiae* Y557 | *S. cerevisiae* MH272-3fα + Lifeact E16R-mCherry[Ade] (2482) | This study |
| **Plasmids for experiments in *S. cerevisiae*** | | |
| 2473 YEpGal555 | *E. coli*/*S. cerevisiae* shuttle vector [ADE2] with Gal1 promoter | (38) |
| 2474 pESC-Ura | *E. coli*/*S. cerevisiae* shuttle vector [URA3, Amp^r^] with Gal1 and Gal10 promoters | Agilent |
| 2475 p1387 pRS313 Actin | *E. coli*/*S. cerevisiae* shuttle vector [HIS3, Ap^r^] based on pRS313 vector, containing Act1 gene with native promoter and terminator. | (35) |
| 2476 pB399 pESC-Ura Lifeact-mCherry | The mCherry gene was amplified from 693 using oligonucleotides tatagaattcatggtgagcaagggcgaggag and tataatcgatgacttgtacagctcgtccatgcc. The resulting PCR fragment was digested with EcoRI and ClaI and ligated into digested 2474 pESC-Ura vector. | This study |
| 2477 pB430 YEpGal555 Lifeact-mCherry | DNA fragments containing 5’UTR and the Lifeact-encoding sequence of ABP140 were amplified from genomic DNA of *S. cerevisiae* MH272-3fα strain using oligonucleotides tatagcggccgcagaactgcaccgtacgctcaga and ctcactggcgcgccttcttcctttgagatgctttcg.  The 3’UTR of ABP140 was amplified from genomic DNA of *S. cerevisiae* MH272-3fα strain using oligonucleotides gataagatctgaaataggaagttctgagacaagtacc and tatagagctcaaattttatatacacgaaggtg. mCherry was amplified from 2476 using oligonucleotides cttcctatttcagatcttatcgtcgtcatcc and ggaagaaggcgcgccagtgagcaagggcgaggaggataac. Products of these three independent reactions were mixed and amplified with oligonucleotides tatagcggccgcagaactgcaccgtacgctcaga and tatagagctcaaattttatatacacgaaggtg. The final PCR product was digested with NotI and SacI and ligated into digested 2473 YEpGal555 vector. | This study |
| 2478 pB449 pRS313 Actin L349M | The L349M mutation was generated by two-step overlap PCR using oligonucleotides tcttggcttctatgactaccttccaa, caggaaacagctatgacc, ttggaaggtagtcatagaagccaaga and ttcgtgataagtgatagtg. The PCR product was digested with ClaI and SalI and was used to exchange the WT ACT1 gene in 2475 p1387 pRS313 Actin. | This study |
| 2481 pB464 pESC-Ura Lifeact-MBP | The plasmid was constructed in two steps. First, LifeAct-coding sequence was added to 2480 by inserting annealed oligonucleotides catgggtgtcgcagatttgatcaagaaattcgaaagcatctcaaaggaagaagg and taccttcttcctttgagatgctttcgaatttcttgatcaaatctgcgacacc into NcoI/NdeI-digested 2480. Then, the resulting plasmid was used to amplify Lifeact-MBP fragment using oligonucleotides ctttaagaaggagagaattcatgggtgtc and tatacggccggtgatgatgatgatgatgattgttg. The PCR product was digested with EcoRI and Eco52I, and ligated into digested 2474 pESC-Ura vector. | This study |
| 2482 pB481 pESC-Ura Lifeact L6K-MBP | The plasmid was constructed in two steps. First, LifeAct-coding sequence was added to 2480 by inserting annealed oligonucleotides catgggtgtcgcagataagatcaagaaattcgaaagcatctcaaaggaagaagg and taccttcttcctttgagatgctttcgaatttcttgatcttatctgcgacacc into NcoI/NdeI-digested 2480. Then, the resulting plasmid was used to amplify Lifeact-MBP fragment using oligonucleotides ctttaagaaggagagaattcatgggtgtc and tatacggccggtgatgatgatgatgatgattgttg, digested with EcoRI and Eco52I, and ligated into digested 2474 pESC-Ura vector. | This study |
| 2483 pB482 pESC-Ura Lifeact F10A-MBP | The plasmid was constructed in two steps. First, LifeAct-coding sequence was added to 2480 by inserting annealed oligonucleotides catgggtgtcgcagatttgatcaagaaagccgaaagcatctcaaaggaagaagg and taccttcttcctttgagatgctttcggctttcttgatcaaatctgcgacacc into NcoI/NdeI-digested 2480. Then, the resulting plasmid was used to amplify Lifeact-MBP fragment using oligonucleotides ctttaagaaggagagaattcatgggtgtc and tatacggccggtgatgatgatgatgatgattgttg, digested with EcoRI and Eco52I, and ligated into digested 2474 pESC-Ura vector. | This study |
| 2484 pB483 pESC-Ura Lifeact I13A-MBP | The plasmid was constructed in two steps. First, LifeAct-coding sequence was added to 2480 by inserting annealed oligonucleotides catgggtgtcgcagatttgatcaagaaattcgaaagcgcctcaaaggaagaagg and taccttcttcctttgaggcgctttcgaatttcttgatcaaatctgcgacacc into NcoI/NdeI-digested 2480. Then, the resulting plasmid was used to amplify Lifeact-MBP fragment using oligonucleotides ctttaagaaggagagaattcatgggtgtc and tatacggccggtgatgatgatgatgatgattgttg, digested with EcoRI and Eco52I, and ligated into digested 2474 pESC-Ura vector. | This study |
| 2485 pB516 YEpGal555 Lifeact L6K-mCherry | A DNA fragment encoding Lifeact with the mutation L6K was created from 2477 using oligonucleotides atatagcgcgccttcttcctttgagatgctttcgaatttcttgatcttatctgcg and tatagcggccgcagaactgcaccgtacgctcaga. Then, the PteI and Eco52I-digested PCR-fragment was used to replace WT Lifeact sequence in 2477. | This study |
| 2486 pB517 YEpGal555 Lifeact F10A-mCherry | A DNA fragment encoding Lifeact with the mutation F10A was created from 2477 using oligonucleotides atatagcgcgccttcttcctttgagatgctttcggctttcttg and tatagcggccgcagaactgcaccgtacgctcaga. Then, the PteI and Eco52I-digested PCR-fragment was used to replace WT Lifeact sequence in 2477. | This study |
| 2487 pB518 YEpGal555 Lifeact I13A-mCherry | A DNA fragment encoding Lifeact with the mutation I13A was created from 2477 using oligonucleotides atatagcgcgccttcttcctttgaggcgctttcg and tatagcggccgcagaactgcaccgtacgctcaga. Then, the PteI and Eco52I-digested PCR-fragment was used to replace WT Lifeact sequence in 2477. | This study |
| 2582 pB521 YEpGal555 LifeAct E16R-mCherry | A DNA fragment encoding Lifeact with the mutation E16R was created from 2477 using oligonucleotides atatagcgcgccttcacgctttgagatgc and tatagcggccgcagaactgcaccgtacgctcaga. Then, the PteI and Eco52I-digested PCR-fragment was used to replace WT Lifeact sequence in 2477. | This study |
| 2583 pB519 YEpGal555 LifeAct E17K-mCherry | A DNA fragment encoding Lifeact with the mutation R17K was created from 2477 using oligonucleotides atatagcgcgcctttttcctttgagatgc and tatagcggccgcagaactgcaccgtacgctcaga. Then, the PteI and Eco52I-digested PCR-fragment was used to replace WT Lifeact sequence in 2477. | This study |
| **Plasmids for protein expression in *E. coli*** | | |
| 1315 pTriEx4_NMHC2C | The motor domain of non-muscular myosin-2C (MYH14, isoform 2 from *H. sapiens*) consisting of amino acids 1–799 | (32) |
| 1609 pET19 tropomyosin | The human tropomyosin alpha-1 gene was synthesized by General Biosystems and cloned into pET19 vector digested with NcoI/BamHI. | This study |
| 1855 cofilin | Human cofilin-1 | (56) |
| 2479 pB386 pET28a MBP-His-ExoY | The ExoY gene was amplified from pUM460 (34) using oligonucleotides tatagagctctggtcgtatcgacggtcatcgtca and tataaagcttcagaccttacgttggaaaaagtc. The resulting PCR product was digested with SacI and HindIII and insterted into pB137 (35) in frame with MBP tag. | This study |
| 2556 pB405 pET28a Lifeact-mCherry-His | The mCherry gene was amplified from 2476 pB399 pESC-Ura Lifeact-mCherry using oligonucleotides ggcgtggccgacctgatcaag and tataggatccgtgatgatgatgatgatgcttgtacagctcgtccatgcc. The resulting PCR product was digested with BamHI and inserted into pET28a digested with NcoI, filled in 5’ overhangs and digested with BamHI. | This study |
| 2557 pB621 pET28a Lifeact_E16R-mCherry-His | The E16R mutation was introduced by PCR with oligonucleotides atataccatgggcgtggccgacctgatcaagaagttcgagagcatcagcaagagggagggggac and cctcccagcccatggtcttcttct and 2556 pB405 pET28a as the matrix. The resulting PCR product was digested with NcoI and exchanged with the bigger fragment of NcoI-digested 2556 pB405 pET28a. | This study |
| 2558 pB622 pET28a Lifeact_E17K-mCherry-His | The E17K mutation was introduced by PCR with oligonucleotides atataccatgggcgtggccgacctgatcaagaagttcgagagcatcagcaaggagaagggggacatg and cctcccagcccatggtcttcttct and 2556 pB405 pET28a as the matrix. The resulting PCR product was digested with NcoI and exchanged with the bigger fragment of NcoI-digested 2556 pB405 pET28a. | This study |
| 565 pET19b-TcdA1 | 6x His-tagged TcdA1 under control of a T7 promoter | (57) |
| 613 pET28a-TcdB2-TccC3 | 6x His-tagged TcdB2-TccC3 fusion under control of a T7 promoter | (43) |
| 579 pET19b-TccC3HVR | 6x His-tagged TccC3(679–960) under control of a T7 promoter | (42) |
| **Plasmids for protein expression in mammalian cells** | | |
| 693 pDEST Lifeact-mCherry | WT Lifeact-mCherry under a CMV promoter | (58) |
| 2492 pB514 pDEST Lifeact F10A-mCherry | To obtain the F10A mutation in Lifeact, the SalI/BamHI fragment of 693 containing WT Lifeact was replaced by a double stranded DNA fragment that was generated by annealing oligonucleotides tcgactggatcatgggcgtggccgacctgatcaagaaggccgagagcatcagcaaggaggagtcgagatatctagacccagctttcttgtacaaagtggttcgatgg and gatcccatcgaaccactttgtacaagaaagctgggtctagatatctcgactcctccttgctgatgctctcggccttcttgatcaggtcggccacgcccatgatccag. | This study |
| 2493 pB515 pDEST Lifeact E17K-mCherry | To obtain the E17K mutation in Lifeact, the SalI/BamHI fragment of 693 containing WT Lifeact was replaced by a double stranded DNA fragment that was generated by annealing oligonucleotides tcgactggatcatgggcgtggccgacctgatcaagaagttcgagagcatcagcaaggagaaatcgagatatctagacccagctttcttgtacaaagtggttcgatgg and gatcccatcgaaccactttgtacaagaaagctgggtctagatatctcgatttctccttgctgatgctctcgaacttcttgatcaggtcggccacgcccatgatccag. | This study |
| mCherry-Actin-C-18 | mCherry-actin under a CMV promoter | (59) |

Supplementary references

55. Peisker K, Braun D, Wolfle T, Hentschel J, Funfschilling U, Fischer G, et al. Ribosome-associated complex binds to ribosomes in close proximity of Rpl31 at the exit of the polypeptide tunnel in yeast. Mol Biol Cell. 2008 Dec;19(12):5279–88.

56. Hsiao JY, Goins LM, Petek NA, Mullins RD. Arp2/3 complex and cofilin modulate binding of tropomyosin to branched actin networks. Curr Biol. 2015 Jun;25(12):1573–82.

57. Gatsogiannis C, Merino F, Roderer D, Balchin D, Schubert E, Kuhlee A, et al. Tc toxin activation requires unfolding and refolding of a β-propeller. Nature [Internet]. Nature Publishing Group; 2018 Sep 19;563(7730):209–13. Available from: http://www.nature.com/articles/s41586-018-0556-6

58. Smyth JW, Vogan JM, Buch PJ, Zhang S-S, Fong TS, Hong T-T, et al. Actin cytoskeleton rest stops regulate anterograde traffic of connexin 43 vesicles to the plasma membrane. Circ Res. 2012 Mar;110(7):978–89.

59. Rizzo MA, Rizzo MA, Davidson MW, Davidson MW, Piston DW, Piston DW. Fluorescent Protein Tracking and Detection: Fluorescent Protein Structure and Color Variants. Cold Spring Harbor Protocols [Internet]. 2009 Dec 1;2009(12):pdb.top63–3. Available from: http://www.cshprotocols.org/cgi/doi/10.1101/pdb.top63
