# Supplementary figures and images for: Structure of the Lifeact–F-actin complex
Source: PLoS Biol. 2020 Nov 20;18(11):e3000925. doi: 10.1371/journal.pbio.3000925 (PMC7717565; doi:10.1371/journal.pbio.3000925)

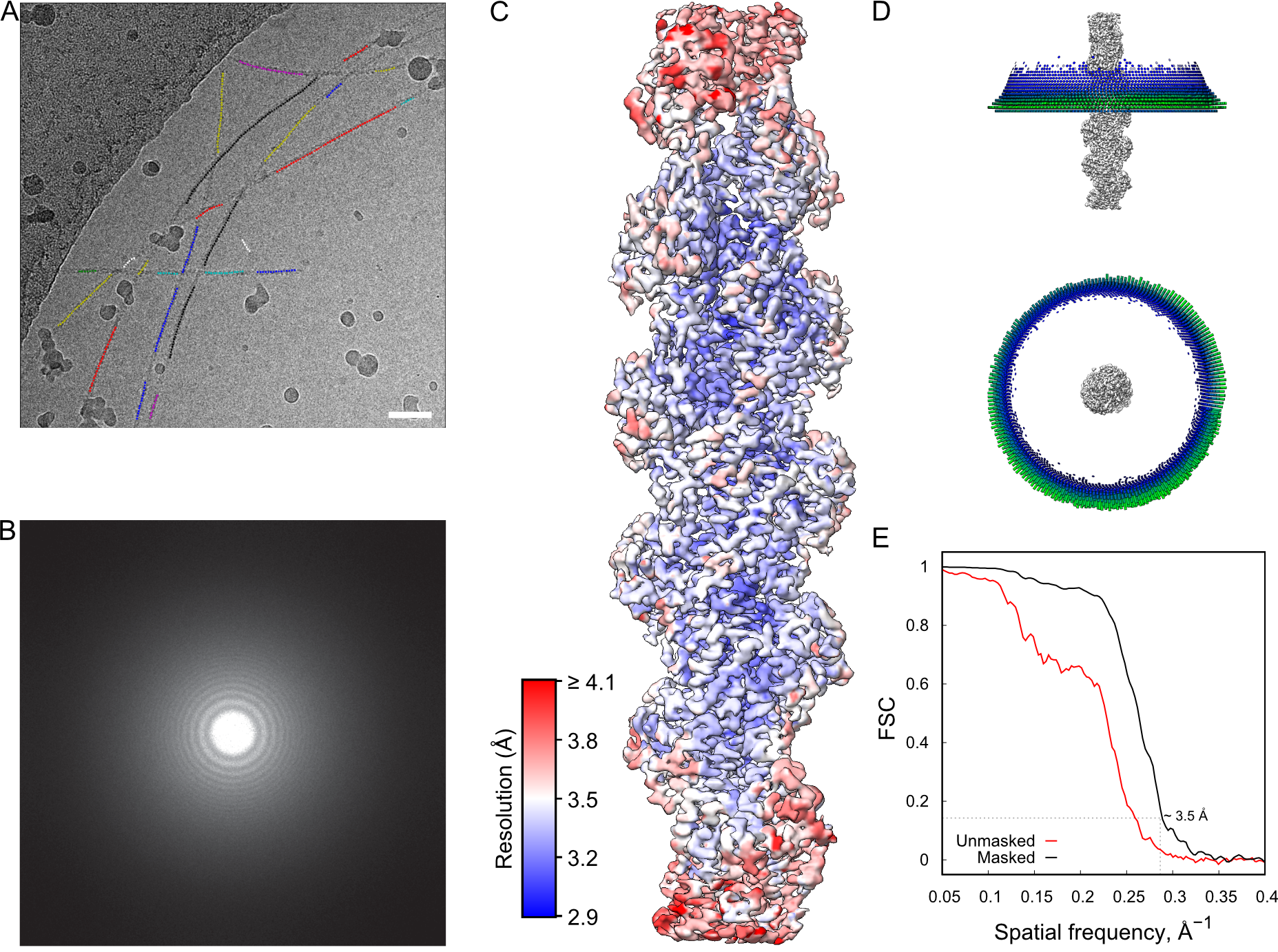

Supplement: S1 Fig — (A, B) Example micrograph (A) and its power spectrum (B) at approximately −1.5-μm defocus. Filaments selected automatically by crYOLO are highlighted as differently colored dots. Scale bars, 10 μm. (C) Density map of the Lifeact–F-actin complex colored according to the local resolution. (D) Orientation distribution of the particles used in the final refinement round. (E) Fourier shell correlation (FSC) for the masked and unmasked final reconstructions. The FSC was calculated in the central 120 Å area of the map. Data points that were used to create this graph are reported in S2 Table. (TIF) [file pbio.3000925.s004.tif]

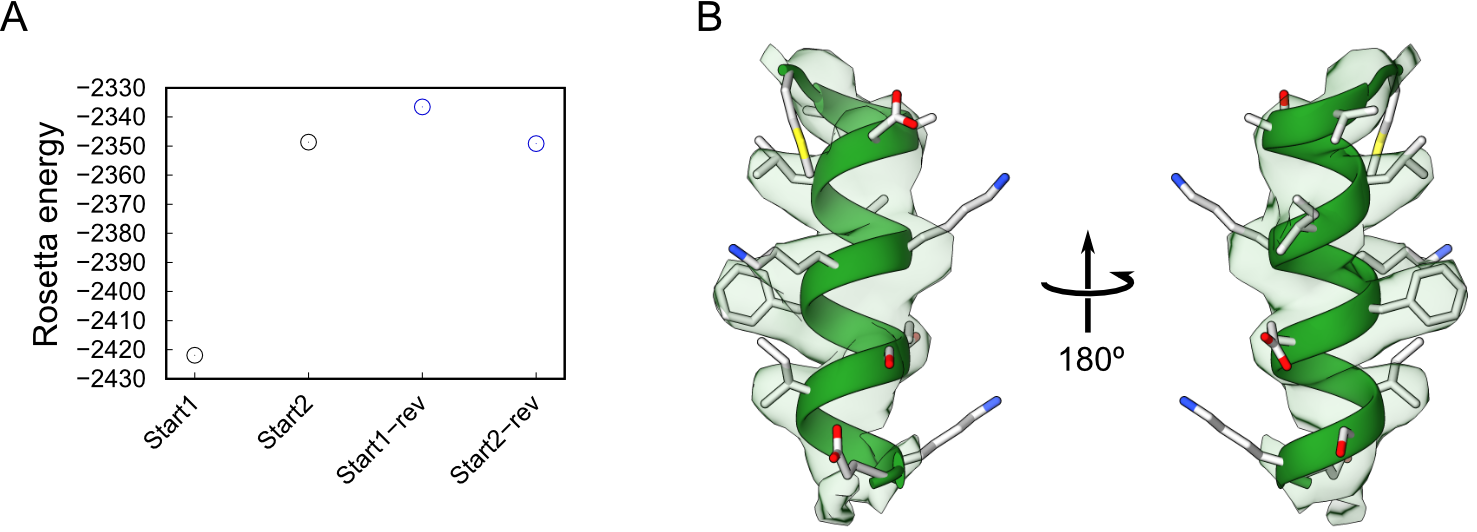

Supplement: S2 Fig — (A) Minimized energy of models of all possible registers of the Lifeact sequence into the density. Start1 and 2 correspond to the peptides starting at M1 or G2 with their N-termini pointing toward the pointed end of the filament. For Start1-rev and Start2-rev, the N-termini points toward the barbed end. The Rosetta energy values show a clear preferred solution. Data points that were used to create this graph are reported in S2 Table. (B) Density fit of the final model corresponding to the energy minimum seen in (A). (TIF) [file pbio.3000925.s005.tif]

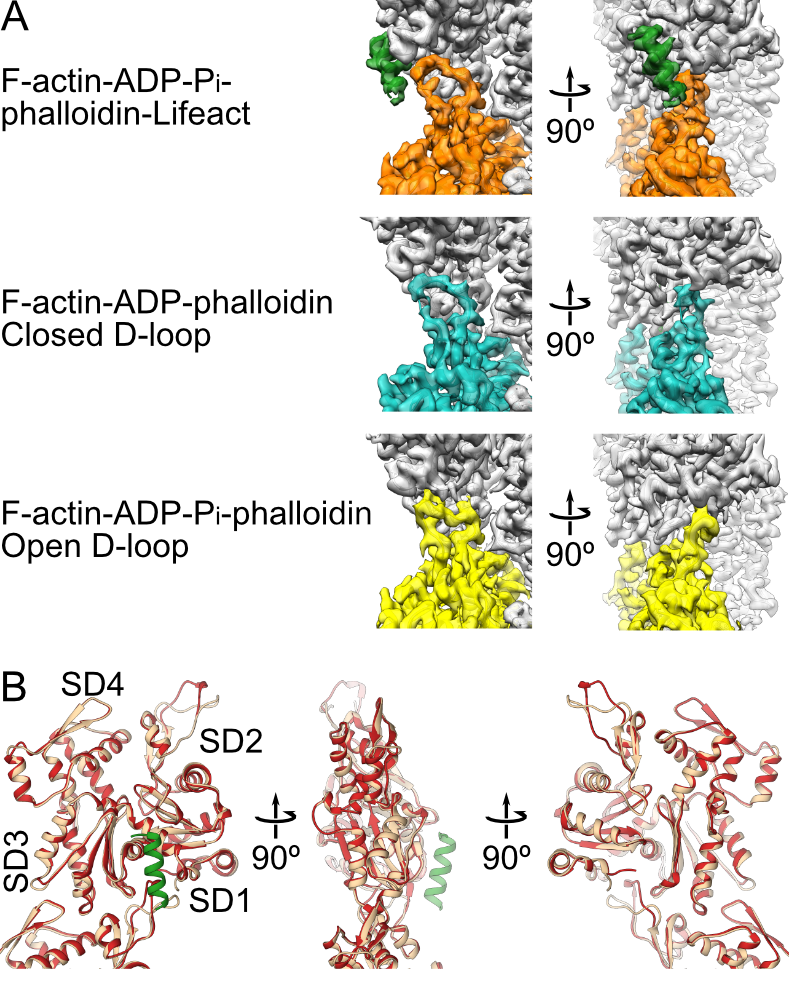

Supplement: S3 Fig — (A) The density maps and corresponding atomic models of Lifeact–F-actin–ADP–Pi–phalloidin in comparison to those of the open D-loop state in F-actin–ADP–Pi–phalloidin (PDB 6T1Y, EMDB 10363) [7] and closed D-loop state in F-actin–ADP–phalloidin (PDB 6T20, EMDB 10364) [7]. (B) Overall comparison of the atomic models of Lifeact–F-actin–ADP–Pi–phalloidin (red) and F-actin–ADP–Pi–phalloidin (beige, PDB 6T1Y, EMDB 10363) [7]. SD, subdomain. (TIF) [file pbio.3000925.s006.tif]

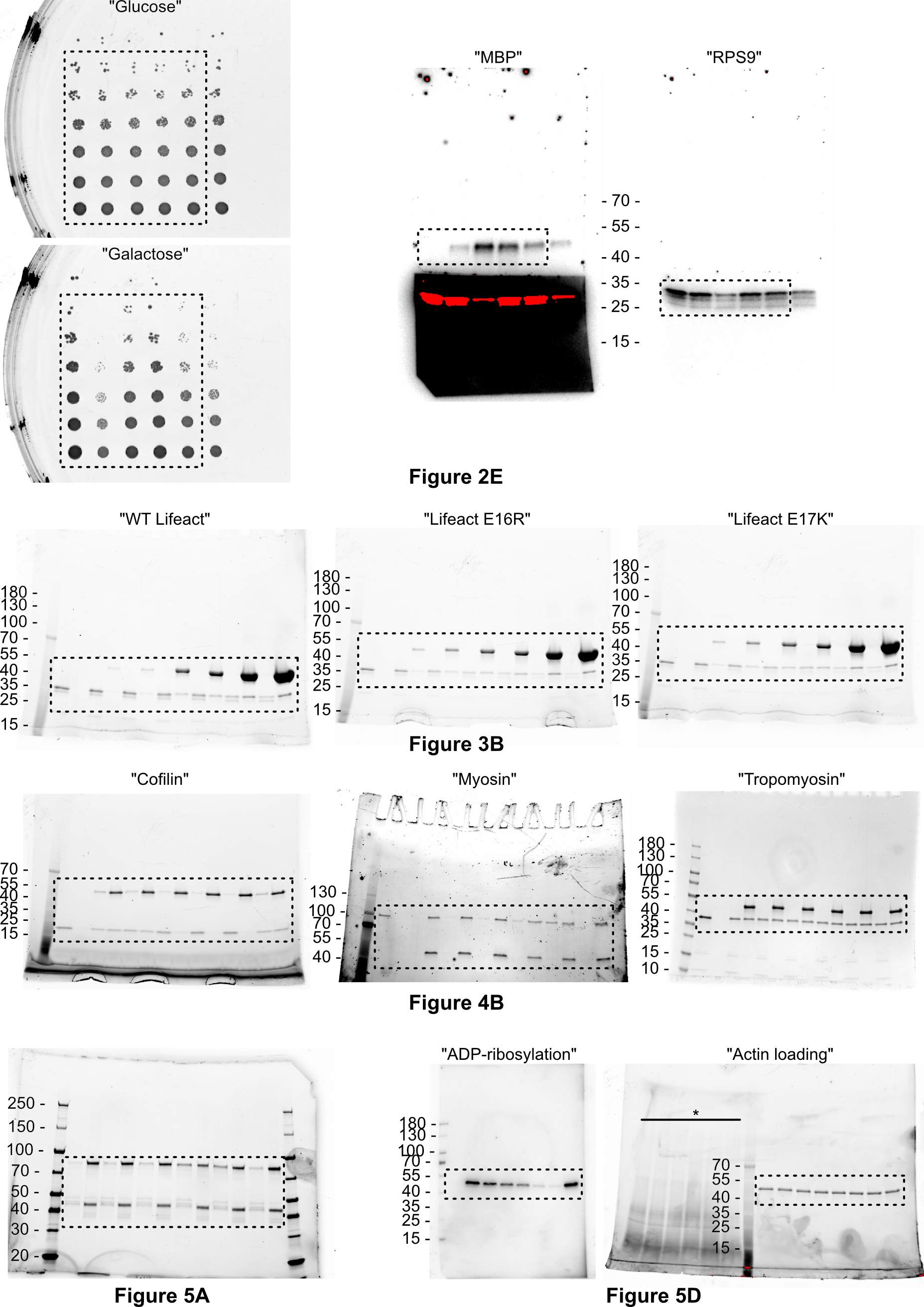

Supplement: S4 Fig — *—This part of the gel was transferred onto PVDF membrane and stained with anti-MBP and anti-RPS9 serum. These western blots are presented on Fig 2E. (TIF) [file pbio.3000925.s007.tif]
