# Supplementary material for: Structure of the Lifeact–F-actin complex
Source: PLoS Biol. 2020 Nov 20;18(11):e3000925. doi: 10.1371/journal.pbio.3000925 (PMC7717565; doi:10.1371/journal.pbio.3000925)
Supplement: Attachment — Submitted filename: Response_to_reviewers.docx [file pbio.3000925.s010.docx]

We thank the reviewers for their constructive and positive feedback, which aided us to further improve the manuscript. Major modifications of the manuscript are highlighted in yellow. Below we include our detailed response to each point.

Reviewers’ comments

In this manuscript, Belyy et al described a cryo-EM structure of Lifact bound actin filament and characterized the likely competition between Lifeact and other actin binding proteins. Lifeact is a widely used probe for actin filaments in vivo. However it has been well-documented that this probe, at a high intracellular concentration, perturbs cellular functions including endocytosis and cytokinesis. Although it has been proposed that Lifeact may interfere with the turnover of actin cytoskeletal structures, the molecular mechanism remained unclear, a question that this study tried to address.

Through this cryo-EM structure, Belyy et al. found that Lifeact, in a helical conformation, interacts with two neighboring actin monomers in a filament. Interestingly, Lifeact interacts specifically with the closed D-loop of one actin monomer. Lifeact competes directly against two essential actin binding proteins cofilin and myosin II to bind actin filaments. Finally, they demonstrated that Lifeact could be employed as a potential antagonist against two bacterial toxins ExoY and TccC3 which also bind actin.

Overall, the well-written manuscript provides novel insights into the interaction between Lifeact and actin filament both in vitro and in vivo. It will be of great interest to many cell biologists. However, the reviewer has several major concerns which are the following:

1) The reviewer is concerned about the novelty of this work. This will be the second cryo-EM structure of Lifeact-F-actin, after the first one was published about four months ago (Kumari et al. 2019 BioRxiv). Based on the manuscript, these two structures largely agree with each other, leading to many similar conclusions, even though the structure solved by Belyy et al. provides slightly higher resolution than the other one (3.5 vs. 4.2 angstrom). The reviewer would suggest that the authors focus more on describing and discussing the novel findings identified through their structure.

With all due respect, we find the concern on the novelty of our publication to be ungrounded. The structure of Lifeact-F-actin complex was indeed posted by Kumari *et al.* in November 2019 on bioRxiv. Our parallel study was posted on the preprint server and submitted to PLOS Biology 4 months later. In this timeframe, the journal considers articles to be contemporaneous and does not evaluate them in terms of novelty. “Results presented in any published or posted contemporaneous articles or preprints by other groups will not impact the editorial assessment or peer review process at PLOS with respect to novelty/strength of advance.” (<https://journals.plos.org/plosbiology/s/complementary-research>). Moreover, until now (19th of June, 2020), the work of Kumari *et al.*, has not yet been accepted by a peer-reviewed journal and the corresponding Lifeact-F-actin structure is not available to the public.

2) The biochemical studies of Lifeact and its mutants need substantial improvements. As an example, the reviewer would question why the authors used 300µM of Lifeact in many of their binding assays. Such high concentration of Lifeact may block nucleation of actin filaments because of its known interaction with actin monomer.

Indeed, to demonstrate the properties of Lifeact we used a concentration that is likely to be above physiological. However, the concern that Lifeact may block nucleation is not relevant in this case, as we used pre-formed F-actin filaments in all our assays.

As another example, the binding assay between actin filament and tropomyosin in the presence of Lifeact (Fig. 4H) was not well designed. Only one concentration (300µM) of Lifeact was used to compete against tropomyosin. More intermediate concentrations of Lifeact, as shown in Fig. 4C and 4E, shall be included to demonstrate that Lifeact doesn't block tropomyosin from binding to actin filaments.

As requested by the reviewer, we performed a new series of co-sedimentation competition experiments with tropomyosin and Lifeact. As expected, lower concentrations of the peptide did not affect the tropomyosin-F-actin interaction (Figure 4B).

3) The in vivo studies of Lifeact and its mutants are not very convincing. For example, the micrographs of actin cytoskeletal structures in budding yeast (Fig. 2A) are of poor quality. Of the three prominent actin structures in budding yeast, only the patch is visible even though the actin cables and the contractile ring shall have been identifiable as well.

Thank you for pointing this out. We agree that the previously presented micrographs showed only patch structures and were therefore not very convincing. In the revised version of the manuscript we present reference examples of yeast cells at different phases of division where all three actin structures are identifiable (Figure 2A,C).

Another example is the quantification of the localization of Lifeact or its mutants in actin patches (Fig. 2B). It would have been much more convincing to examine this using time-lapse microscopy of the actin patches. Through this, it would be possible to quantify the localization of Lifeact mutants in the patch.

To perform colocalization experiments we needed to use phalloidin as a positive control. However, this drug strongly affects the actin cytoskeleton and cannot be used in time-lapse microscopy. Therefore, we can unfortunately not perform the suggested experiment to quantify the localization of Lifeact mutants in the patch using time-lapse microscopy. However, we believe that the new clearer set of yeast images together with the previously measured colocalization coefficients (p values < 0.0001) are convincing enough to prove our hypotheses.

Lastly, the authors failed to provide any evidence that the two novel Lifeact variants (Fig. 3) are better probes than the wild-type Lifeact. A in vivo experiment that uses these two variants E16R and E17K to stain actin cytoskeletal structures could easily test this hypothesis.

We have not claimed that these two variants are better *in vivo* probes than the wild-type. We designed E16R and E17K mutations and tested them *in vitro* as a proof that the affinity of the peptide can be in principle modulated.

However, following the request of this reviewer, we have tested the behavior of the two new Lifeact variants in yeast. In order to do this, we expressed the new Lifeact-mCherry fusions in yeast and observed Lifeact staining of F-actin by confocal microscopy *in vivo* (Figure 2A). To test, whether the introduced mutations made Lifeact a better probe, we performed two series of analysis: Firstly, we calculated the ratio of Lifeact-mCherry to phalloidin fluorescence signal in patches (Fig. 3D) in order to determine if the increased affinity of Lifeact to F-actin indeed increased amount of the fusion protein localized in the F-actin rich patch. Secondly, to estimate the signal-to-noise ratio, we calculated the ratio of the Lifeact fluorescence signal in the patch to the Lifeact signal in the middle of the cell where no phalloidin staining was observed (Fig. 3E). In both analyses, we saw only an insignificant increase of the average signal ratios of the new variants (Fig. 3D, E). A reason for not seeing a stronger difference could be the high affinity of the probe to G-actin which masks F-actin staining (Riedl et al., 2008). We plan a follow-up study to design Lifeact variants with lower affinity to G-actin that will hopefully result in a better signal-to-noise ratio.

4) Some key experiments lack proper quantification. Most importantly, the binding affinity between Lifeact/Lifeact mutants and actin filaments shall have been quantified (Fig.3C-D) and compared to many previous studies (Riedle 2008 and Courtemanche 2016).

As requested by the reviewer, we performed series of co-sedimentation experiments and quantified the binding affinity between Lifeact variants and the actin filaments. In order to compare our affinity numbers with those previously reported, we changed the carrier protein from maltose-binding protein to mCherry that was used by Courtemanche et al., 2016. We obtained comparable K_d_ values to the previously reported (14.9 ± 1.6 µM vs 13.2 ± 0.7 μM).

In another example, no quantitative evidence is provided to support the claim that Lifeact doesn't modify the structure of actin filament. A more detailed comparison between the structure of Lifeact bound actin filament and that of unbound actin filament (Oda et al. 2009), will be highly informative.

We believe that our 3.5 Å resolution structural model provides convincing evidence to support the claim that Lifeact does not alter the structure of actin filament. In order to address the referee’s concern and better illustrate our finding, we added a figure with a comparison of our previous high-resolution F-actin-phalloidin structures with our Lifeact-F-actin-phalloidin structure (Figure S3B).

Minor concerns:

1) Fig. 1: It would be important to provide an overview on which subdomains of the actin monomer, from SD1 to SD4, interact with Lifeact.

We have modified the figure as requested.

2) Fig. 2C: It is not clear from the text whether the endogenous actin had been replaced with the two actin mutants (D25Y and L349M), an important condition to interpret this result.

We have corrected the text and figure legends as requested.

3) Fig. 3D: The binding between the actin filament and wild-type Lifeact never reached a plateau, which makes it hard to estimate the affinity between them. Higher concentrations of Lifeact shall be used to saturate the filaments.

Answering to the reviewer’s major concern 4, we performed a new series of co-sedimentation experiments and quantified the binding affinity between Lifeact variants and the actin filaments (Fig 3B, C). As the reviewer suggested to compare our results with the previously published, we needed to adjust our experimental setups. For example, we changed the carrier protein from maltose-binding protein to mCherry as it was done by Courtemanche *et al.*, 2016. For the same reason, we decided not to use higher Lifeact concentrations, as the reviewer suggested, because in the publication of Courtemanche *et al.*, 2016, the authors used 2 μM LifeAct–mCherry. Nevertheless, we believe that higher concentrations of F-actin that we used on the Fig. 3C allowed us to reliably measure K_d_ values of Lifeact variants. The curve shape and the measured K_d_ values are consistent with the published data (Courtemanche et al., 2016).

4) Fig. 5A-B: The representative gel picture is less than ideal. A smudge obscured part of the lane where the supernatant for 300 µM Lifeact was. Please replace it.

We replaced the gel picture as requested by the reviewer.

5) Fig. 5F: The plot is difficult to understand. It is highly preferable to replace it with time courses of average value ± standard deviation at each data point.

We changed the graph as requested by the reviewer. However, we think that presenting all experimental points is preferable to average values in terms of data transparency. Therefore, to make the plot easier to read, we split it into four subplots positioned next to each other.

6) Please scale back the statement that Lifeact compete with the bacterial toxins to bind actin filament. The study provided no evidence that Lifeact competes directly with either of the bacterial toxins to bind actin filaments. The inhibitory effect of Lifeact to Tcc3HVR and ExoY in vivo could be indirect.

We kindly disagree with this and have reason to believe that our statement is correct, because:

1) TccC3HVR modifies Thr148 (Lang et al., Science 2010) which is positioned within a 4 Å distance of Lifeact’s Phe10;

2) A mutation in Asp25 of actin abolishes its binding to ExoY (Belyy et al., Nat Commun 2016; Belyy et al., JBC 2018). At the same time, Asp25Tyr also impairs Lifeact-F-actin complex formation.

In order to convince the reviewer and provide more detailed information to the readers, we added the Lifeact-F-actin structure with marked Thr148 and Asp25 to Figure 5C.

7) Please clarify which myosin the authors referred to throughout the main text and figure legends. The reviewer assumed that it is non-muscle myosin II.

The reviewer is correct regarding the type of myosin (2C), which is specified both in the main text and in the material and methods sections. We have now additionally added this information to the figure legends.

In summary, this study by Belyy et al. has the potential to make a significant contribution to our understanding of Lifeact but it requires major revisions. The reviewer will be happy to review the revised manuscript when it becomes available.

We thank the reviewer for the comprehensive and positive feedback to our manuscript. We hope that both our replies here and corrections in the revised manuscript have helped to alleviate the concerns raised.

Rev. 2:

Summary

This is a timely and important paper that describes new observations on a widely used actin-binding probe molecule, including a high-resolution co-complex with actin filaments. Key observations from the structure include the prediction that Lifeact binding interferes with binding of key cytoskeletal cofactors including myosin and cofilin, as well as bacterial toxins. These predictions are borne out by biochemical studies and in vivo studies where they show that Lifeact competes with myosin and cofilin and interferes with bacterial toxin activity.They further modify Lifeact properties with structure-guided design, supported by binding measurements. The structure presented here also provides additional evidence to support prior predictions by this group that actin's key 'D-loop' element can adopt discrete 'open' and 'closed' conformations, whose equilibria are potentially affected by ATP hydrolysis and cofactor binding .

Overall Sentiment

This paper is well written and succinct. I have no reservations to publish it immediately. Understanding the molecular basis of Lifeact-F-actin binding will enable researchers to use it with greater prudence and sophistication.

Major concerns

None

Minor concerns

The two sentences of the results/discussion are a little off, although the intended meaning can be gotten from them. I think the message that is intended is that (1) prior studies have indicated a molecular basis for stabilization of actin filaments by phalloidin (2) prior studies indicate phalloidin places actin in a well defined nucleotide state with a well-defined D-loop conformation, making it a suitable reference state for structure studies with Lifeact.

We thank the reviewer for the positive feedback on our manuscript. We have changed the sentences as suggested.

Rev. 3:

Lifeact is a chemically modified 17-mer peptide derived from the N-terminal end of the methyltransferase ABP140, an actin-binding protein able to mediate actin localization. It is today one of the most versatile live-cell actin tools with which to visualize actin dynamics in the cell. Genetically encoded actin reporters, either fluorescent derivatives of actin or actin-binding domains, although powerful tools for studying actin filament architecture and dynamics in live cells, are biased probes and their cellular distribution does not accurately reflect that of the cytoskeleton. The differences in live-cell actin probe localization can be associated to efficiency of incorporation capacity, competition with endogenous actin associated proteins, and differential rates of actin filament turnover. In addition to their fidelity with which they reveal the architecture of the actin cytoskeleton, special attention to establishing expression levels that do not alter cell morphology

or behavior is required.

Biochemical and structural studies defining the mechanisms underlying the actin binding interactions of actin probes could become powerful tools, providing mechanistic insight into the formation of actin networks in live cells. Here, Raunser and colleagues address this exact point, and thus this work is of importance.

The team presents a 3.5-Å structure of Lifeact / phalloidin-stabilized F-actin complex using single particle cryo-EM. In the reconstruction densities corresponding to ADP, Mg2+ and Pi in the nucleotide-binding pocket of actin and a density corresponding to phalloidin at the expected positions were all identify. Of the Lifeact peptide, 16 out of 17 amino acids were unambiguously fit into the density. Lifeact interacts with a hydrophobic binding pocket on F-actin and stretches over two adjacent actin subunits, stabilizing the DNase I binding loop of actin in the closed conformation. Through actin co-sedimentation assays the authors showed Lifeact competes with cofilin (assuming no F-actin change) and myosin, and suggest that the morphological artefacts described for Lifeact are caused by competition for the same binding site on F-actin. In addition, Lifeact competes with actin-binding toxins such as ExoY and TccC3HVR, demonstrating the potential of Lifeact to serve as a

platform for anti-toxin drugs. The team also employed site directed mutagenesis to provide Lifeact probes with modified properties expanding the functional role of this probe.

One point that requires a bit more rigorous testing: The relevance of the co-sed assays that requires over 100umol of Lifeact to show effect seems a bit puzzling in terms of its relevance to in vivo conditions. One possible solution would be to perform a fluorescence quenching experiment with pre-assembled Lifeact F-actin. Adding myosin or cofilin should show quenching behavior hopefully at less unphysiological concentrations.

We thank the referee for the positive review of our manuscript. We agree that such high Lifeact concentrations are likely to be above physiological levels. To address the reviewer’s concern, we have repeated the co-sedimentation experiments presented in Figure 4.

Particularly for the competition assay with cofilin, we added an experimental point with 10 µM of Lifeact. At this concentration Lifeact fully outcompeted cofilin. Interestingly, a similar concentration of Lifeact inhibited ADP-ribosylation of actin by TccC3HVR *in vitro* (Figure 5). As we observed a protective effect of Lifeact during TccC3HVR intoxication *in vivo*, this Lifeact concentration should therefore be near-physiological.

For the competition with myosin, we changed the experimental setup. First, we added phalloidin to F-actin after its polymerization and not during polymerization. This should mimic the more physiological closed state of the F-actin D-loop and increase affinity of Lifeact to it (Kumari et al. 2019 BioRXiv). Second, we used lower amounts of myosin and F-actin in the experiment to make a non-saturated F-actin-myosin complex. As a result, we measured a 20% decrease of bound myosin in the presence of 30 uM of Lifeact. In our experiments we used a myosin variant with high affinity to F-actin. We hypothesize that *in vivo* modification of myosin or F-actin may decrease this affinity (for example, as modification of actin by transglutaminases, Del Duce et al., Biochem J 2009; or ALKBH4-dependent demethylation of actin, Ming-Ming Li et al., Nat Commun 2013) making it easier to Lifeact to modulate the interaction.
